# A Selective Review of Multi-Level Omics Data Integration Using Variable Selection

**DOI:** 10.3390/ht8010004

**Published:** 2019-01-18

**Authors:** Cen Wu, Fei Zhou, Jie Ren, Xiaoxi Li, Yu Jiang, Shuangge Ma

**Affiliations:** 1 Department of Statistics, Kansas State University, Manhattan, KS 66506, USA; feiz@ksu.edu (F.Z.); jieren@ksu.edu (J.R.); xiaoxili@ksu.edu (X.L.); 2 Division of Epidemiology, Biostatistics and Environmental Health, School of Public Health, University of Memphis, Memphis, TN 38152, USA; yjiang4@memphis.edu; 3 Department of Biostatistics, School of Public Health, Yale University, New Haven, CT 06510, USA; shuangge.ma@yale.edu

**Keywords:** integrative analysis, multi-level omics data, parallel and hierarchical integration, Penalization, Bayesian variable selection

## Abstract

High-throughput technologies have been used to generate a large amount of omics data. In the past, single-level analysis has been extensively conducted where the omics measurements at different levels, including mRNA, microRNA, CNV and DNA methylation, are analyzed separately. As the molecular complexity of disease etiology exists at all different levels, integrative analysis offers an effective way to borrow strength across multi-level omics data and can be more powerful than single level analysis. In this article, we focus on reviewing existing multi-omics integration studies by paying special attention to variable selection methods. We first summarize published reviews on integrating multi-level omics data. Next, after a brief overview on variable selection methods, we review existing supervised, semi-supervised and unsupervised integrative analyses within parallel and hierarchical integration studies, respectively. The strength and limitations of the methods are discussed in detail. No existing integration method can dominate the rest. The computation aspects are also investigated. The review concludes with possible limitations and future directions for multi-level omics data integration.

## 1. Introduction

The advancement of high-throughput technologies has generated unprecedented amount and types of omics data. Comprehensive molecular profiling has been conducted to profile biological samples on different layers of genomic activities including mRNA, microRNA, CNV and DNA methylation [1,2,3]. The emergence of the multi-omics data has brought new challenges to the development of statistical methods for integration.

Traditionally, omics data analysis has been conducted as a single-level manner where joint analysis of omics data from more than one level has not been considered. A representative example is gene expression analysis when microarrays have dominated in the early 2000s. That is perhaps the first time that the development of statistics methods for high dimensional data has been systematically driven by the need of analyzing data from the real world. Consider a data matrix where the columns correspond to variables (i.e., gene expressions) and rows correspond to samples. This data matrix is of the “large data dimensionality, small sample size” nature as in the microarray studies, the number of gene expressions is usually much larger than the sample size. With a clinical outcome such as cancer status or patients’ survival, the central question cancer researchers ask is: “How to select a subset of important gene expressions that is associated with the clinical outcome?” Statistically, omics features, such as gene expressions, are treated as variables in the model. It can be recast as a variable selection problem, where a small set of informative variables needs to be identified out of a large number (or dimension) of candidates. LASSO is one of the most popular variable selection methods for analyzing high dimensional data [4]. It has been developed to select important features while continuously shrinking the regression coefficients of the features to zero. More specifically, when the coefficient is shrunk to 0, the corresponding feature is not included in the selected model. Thus, variable selection has been conducted simultaneously with parameter estimation. The phenomena of 0′s in the estimated coefficients is termed as sparsity in variable selection literatures.

The development of variable selection methods with applications to high-dimensional data especially from biological studies has been one of the most popular research topics in statistics in the past two decades. The unique advantages of variable selection lie in its interpretability (since merely a small number of genes or other omics features will be included in the model) and well-established statistical theories including the oracle properties under high dimensionality [5,6,7]. Moreover, other popular statistical methods for analyzing large scale omics data, such as dimension reduction, can also be viewed and recast as a variable selection method when the sparse counterpart is of interest. For example, principal component analysis (PCA) is a well-known dimension reduction method. Sparse PCA overcomes the drawback of PCA and can be formulated as a variable selection problem [8]. Recently, as the multi-level omics data has become available, a paradigm shift from single level omics data analysis to multi-omics data integration has been observed in the development of variable selection methods, which poses new challenges to computation as generally complicated optimization techniques are involved. We believe that conducting a review on integration methods that have focused on variable selection will provide a novel and fresh perspective to investigate integrative analysis.

**Figure 1:**
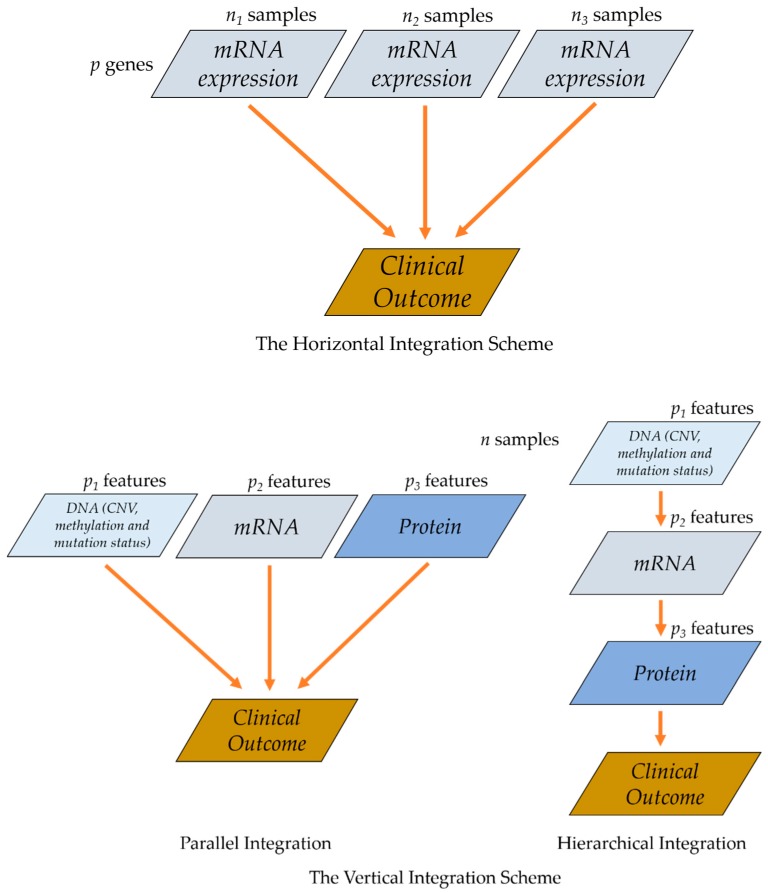
The Horizontal and Vertical Integration Schemes.

The term integration (or integrative, integrated) analysis has been used extensively to mainly describe studies that integrate multiple sources of data in the following two aspects: (1) integrating the same type of omics data across different studies and (2) integrating different types of omics data for the same cohort of samples. For example, meta-analysis combining summary statistics from multiple GWAS studies, and joint analysis of multiple datasets with different cohorts of samples and overlapping omics features based on homogeneous and heterogeneous assumptions (Zhao et al. [9]) are both of the first case. Here we focus on the second scenario. Note that Richardson et al. [10] also refer (1) and (2) as horizontal data integration and vertical data integration. Here we follow the terms for convenience of description and show them in Figure 1. More discussions on Figure 1 will be provided in following sections.

There are multiple reviews on vertical integrative methods for multi-level data, including both comprehensive reviews [10,11,12,13,14,15,16,17] and those focusing particularly on a specific type of integration method, such as clustering analysis [18,19,20,21]. While the comprehensive reviews are invaluable for gaining an overall picture of multi-level omics data integration analysis, a common limitation is the lack of an accurate and unifying framework to summarize integration methods, which seems inevitable given the diversity of large amount of methodological work in this area. On the other hand, reviews with emphasis on a particular type of integration method provide an important addition to the summary of existing multi-level omics integrative analysis [18,19,20,21,22,23,24,25].

This review significantly differs and advances from existing ones. We use the integration strategy as the main criterion to categorize representative multi-dimensional data integration studies, with an emphasis on the variable selection methods developed in these studies. We believe such an arrangement will shed new insight in understanding this area from the angle of variable selection which is one of the most popular tools for integrative analysis. As discussed in this introduction, variable selection has played a key role in analyzing single-level omics data which is usually of high dimensionality. Multi-omics data is the aggregation of single-level omics data. Therefore, integrative analysis on multi-omics data generally demands variable selection techniques. It is worth noting that there are published literatures on summarizing based on the statistical methods in integrative analysis. For example, [18,20,21] are focused on reviewing clustering methods, and Ickstadt et al. [22] weighs more on Bayesian methods. This article distinguishes itself from yet complements published multi-omics integration reviews by prioritizing the role of variable selection according to the structure of integration studies. For convenience, we summarize existing reviews on integrating multi-level omics data in Table 1. We also acknowledge that our review is methodologically oriented. Please refer to Hasin et al. [12], Rendleman et al. [24] and studies alike for reviews focusing on biological and other aspects of multi-omics data integration.

The rest of this article is organized as follows. We describe the variable selection methods, including penalization and Bayesian variable selection that have been extensively adopted for integrative analysis. Then, we discuss the integrative analyses that have been performed in the supervised, semi-supervised and unsupervised manner, within both parallel and hierarchical integration studies. The strength and limitations of the reviewed methods have been discussed. We have also investigated the computational algorithms of variable selection methods in multi-omics integration. Finally, we conclude with discussions and possible extensions.

## 2. Statistical Methods in Integrative Analysis

Most of the statistics methods for the vertical integration of multi-dimensional omics data are model based and can be characterized as regression analysis (supervised) or exploratory analysis (unsupervised), depending on whether the aim of study is prediction under phenotype traits (such as disease phenotype, cancer status and cancer survival) or not (Richardson et al. [10]). In both types of analysis, separating noisy signals from important ones plays a crucial role. In regression analysis, identifying the subset of relevant omics features from a large number of candidates leads to improved prediction and better interpretation of the selected model. On the other hand, in the exploratory analysis, even without an explicit clinical outcome, such as disease phenotype or patients’ survival, sparse identification of loading vectors is still appealing. For example, canonical correlation analysis (CCA) is an ideal dimension reduction method to integrate a pair of omics datasets (Meng et al. [23]). A sparse version is essential for CCA applicable for high dimension low sample size scenario (Witten et al. [26]). In principal component analysis based study, such as JIVE (Lock et al. [27]), sparse estimation on variable loadings has been pursued to achieve better interpretation and understanding of the multi-level omics integration results.

Therefore, we take an alternative route to summarize the model based methods from the perspective of variable selection, which has not been conducted in published reviews on integrative analysis. It will shed new insight on how the multi-omics data motivate methodological development in integration studies. We acknowledge that there exists a diversity of integrative methods, including non-model based methods (Richardson et al. [10]) as well as graph/network based methods (Yan et al. [25]). In this paper, we focus on reviewing integrated studies performed by using variable selection methods. A brief overview of the methods is provided below.

### 2.1. Penalized Variable Selection

Penalization or regularization, is perhaps the most popular framework to select important omics features in multi-dimensional integration studies. Consider disease outcome *Y*, where *Y* can be a continuous disease phenotype, categorical cancer status or survival of patients. *X* is the design matrix denoting the *p*-dimensional genomics features such as SNPs, DNA methylation and gene expressions, among other omics measurements for *n* subjects. A central task in analyzing the “large *p*, small *n*” or high dimensional genomics data is to identify important features that are associated with disease phenotype, from a large set of candidate features. The modelling can be formulated as follows:

(1) $$\hat{\beta }={\mathrm{argmin}}_{\beta }\left\{L\left(\beta ;Y,X\right)+\mathrm{pen}\left(\beta ;\lambda \right)\right\}$$

where *L*(•) is the loss function measuring the lack of fit of the model and pen(•) is the penalty function controlling the sparsity of the model through the data-dependent tuning parameter λ. The penalty function imposes shrinkage on the coefficient vector β corresponding to the high dimensional genomics features. $\beta_{j}$ is the coefficient corresponding to the jth omics feature. Variable selection can be achieved ($\beta_{j}$ = 0) simultaneously with penalized estimation. LASSO is of the form of “least square loss+ L1 norm”, that is, $|\left|Y-X\beta \right|{|}_{2}^{2}+\lambda \left|\beta \right|$, where $\lambda \left|\beta \right|=\lambda \sum_{j=1}^{p}\left|\beta_{j}\right|$. It is among the family of baseline penalization methods such as adaptive LASSO [6], SCAD [5] and MCP [28]. Popular choices of the penalty functions in multi-omics integration studies, as shown by our review, include LASSO [4], elastic net [29] ($\lambda_{1}|\left|\beta \right|{|}_{2}^{2}+\lambda_{2}\left|\beta \right|$) and fused LASSO [30] ($\lambda_{1}\sum_{j=1}^{p}\left|\beta_{j}\right|+\lambda_{2}\sum_{j=2}^{p}|\beta_{j}-\beta_{j-1}|$), among many other penalty functions. For more detailed and comprehensive review on variable selection and its applications in bioinformatics studies, please refer to Ma and Huang [31], Fan and Lv [7] and Wu and Ma [32].

### 2.2. Bayesian Variable Selection

Bayesian variable selection is a classical statistical method for analyzing high dimensional data. It can be classified into the four categories (1) adaptive shrinkage, (2) indicator model selection, (3) stochastic search variable selection and (4) model space approach [33]. With the cancer genomics data, Bayesian methods have found new applications [22], especially for the adaptive shrinkage [34,35,36] and indicator model selection method (including stochastic search variable selection), such as [37,38,39].

Tibshirani [4] has examined LASSO from a Bayesian perspective. The LASSO estimate can be viewed as the posterior estimate when independent and identical Laplace prior has been imposed on regression coefficient $\beta_{j}$ (*j* = 1, …, *p*):

(2) $$p\left(\beta_{j}|\tau \right)=\frac{1}{2\tau }e^{-\left|\beta_{j}\right|/\tau }$$

with τ=1/λ. Bayesian LASSO ([34]) has been proposed by specifying a conditional Laplace prior on $\beta_{j}$

(3) $$p\left(\beta_{j}|\sigma^{2}\right)=\frac{\lambda }{2\sqrt{\sigma^{2}}}e^{-\lambda \left|\beta_{j}\right|/\sqrt{\sigma^{2}}}$$

Conditioning on $\sigma^{2}$ guarantees unimodality of the posterior distribution [34]. As LASSO belongs to the family of shrinkage estimate induced by $L_{q}$ norm under *q* = 1, the other shrinkage estimates from this family can also be interpreted as Bayesian estimates with different priors, which significantly enriches the adaptive shrinkage methods for Bayesian variable selection. The rational of specifying the prior has also been carried over to other LASSO type of penalization methods, including fused LASSO, group LASSO and elastic net (Kyung et al. [40]). One disadvantage of Bayesian LASSO is that the penalized estimate cannot achieve 0 exactly, which has been overcome in multiple studies through, for instance, introducing spike-and-slab priors ([41,42,43]) which have the following form:

(4) $$\beta_{j}|\gamma_{j}\;\begin{array}{c} ind\\ \sim \end{array}\;\gamma_{j}\phi_{0}\left(\beta_{j}\right)+\left(1-\gamma_{j}\right)\phi_{1}\left(\beta_{j}\right),\;j=1,\ldots ,p$$

where $\gamma_{j}\in \left\{0,1\right\},\;\phi_{0}\left(\beta_{j}\right)$ denotes a spike distribution for zero coefficients corresponding to unimportant effects and $\phi_{1}\left(\beta_{j}\right)$ denotes a slab distribution for large effects. Note that there are other priors of a similar two-component form, such as the two-component g prior [44]. They can also be used for variable selection purposes.

Indicator model selection, on the other hand, conducts variable selection based on a mixture prior density with a latent indicator vector $\theta \in {\left\{0,\;1\right\}}^{p}$. Whether or not the jth predictor is included in the model is corresponding to $\theta_{j}=1$ and $\theta_{j}=0$, respectively. For example, with the spike-and-slab prior, $\beta_{j}$ can be set as 0 in the spike component, which is corresponding to $\theta_{j}=0$. The indicator prior can be modified to incorporate informative biological information, such as gene set, pathways and networks, in the selection procedure [45,46,47].

**Remarks on Other Variable Selection Methods:** Here we focus on penalization and Bayesian variable selection since the two have been the primary variable selection methods adopted for multi-level omics studies reviewed in this paper. In addition, there exists a diversity of variable selection methods that are also applicable in the integrative analysis. For example, popular machine learning techniques include random forest and boosting. In random forest, the variable importance measure can be adopted to conduct variable selection [48]. Boosting is a strong learner based on an ensemble of multiple weak learners, such as individual gene expression, CNV and other omics features. Within the linear regression setting, boosting selects variables having the largest correlation with residuals corresponding to the current active set of selected predictors (the weak learners) and move its coefficient accordingly. The prediction power has improved significantly in boosting through aggregating multiple weak learners [49].

**Remarks on Connections among Integrative Analysis, Variable Selection and Unsupervised Analysis:** Variable selection has been widely adopted for analyzing the single level omics data where the dimensionality of omics features is generally much larger than the sample size. Identification of a subset of important features usually leads to (1) better interpretability and (2) improved prediction using the selected model. The two are also critical for the success of integrative analysis of multi-omics data. This fact at least partially explains why variable selection is among one of the most powerful and popular tools for data integration. Even for integration studies that do not use feature selection explicitly, as we discuss in following sections, a screening procedure is generally adopted to reduce number of features before integration.

The formulation of variable selection problems also shed insight on the interaction between itself and integrative analysis. Penalization has the form of “unpenalized loss function + penalty function”, where the unpenalized loss function is characterized by the nature of integration and the penalty function determines how the selection is conducted. With the loss function, the choice of penalty functions is not always arbitrary. For example, the least absolute deviation loss has L1 form, so penalty functions based on L1 norm, such as LASSO and fused LASSO, are computationally convenient. Penalty functions involving quadratic terms, such as network-constrained penalty [50], work well with quadratic loss function [51] but they need to be approximated by a surrogate in the form of L1 norm to reduce computational cost [52]. Therefore, for integrative analysis, the nature of integration does have an impact on the way that the variable selection is conducted.

Unsupervised techniques, such as PCA, CCA, PLS and clustering analysis, can be viewed as optimization problems with different objective functions. For example, principal component analysis can be reformulated as a ridge regression with the normalized regression coefficients denoting PC loadings. The objective function is in a least square form measuring the approximation loss involving the PCs [8]. Besides, CCA and PLS investigate the relation between two groups of variables by maximizing the correlation and covariance between the two sets of variables, respectively, where the loadings are optimization variables of interest [53,54]. In addition, K-means clustering is a popular method for conducting clustering analysis and can be viewed as minimization over within-cluster sum of squares (WCSS). Overall, the characteristics of these unsupervised methods are reflected in the corresponding loss (or objective) functions.

For even dealing with single level high dimensional omics data, the sparse properties of the unsupervised methods are attractive. Sparse unsupervised techniques have already been developed for single-level omics data analysis and their connections to penalization are well-established. For example, Zou et al. [8] has shown the equivalence between sparse PCA and regularization, which uses elastic net to yield modified principal component with sparse loading vectors. Witten and Tibshirani [55] has developed the sparse K-means and sparse hierarchical clustering using LASSO to conduct feature selection in clustering. Besides, Witten and Tibshirani [26] and Lê Cao et al. [56], among many other studies, have investigated sparse CCA and sparse PLS as a penalization problem, respectively. The importance of variable selection naturally carries over from single platform based analysis to multi-omics integration studies. Extensive sparse unsupervised techniques have been developed and applied for analyzing multi-level omics data.

**Figure 2:**
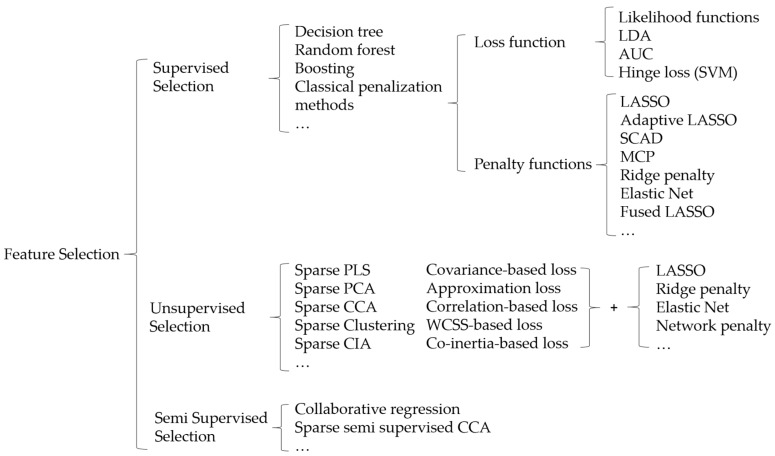
A taxonomy of variable selection in supervised, unsupervised and semi supervised analyses.

Overall, the optimization criterion, which is formulated as “unpenalized loss function + penalty function” provides a new perspective of investigating integrative analysis, especially the interactions between integrating multi-omics data and omics feature selection. Based on this formulation, Figure 2 shows a taxonomy of variable selection in terms of the supervised, unsupervised and semi-supervised manner in multi-omics data integration studies. We acknowledge that such a summary can hardly be exhaustive even for integration studies themselves. So “…” denotes that there are other studies not on the list.

## 3. Multi-Omics Data Integration

The high dimensionality of multi-level omics data is two folded. First, each type of omics measurement (such as mRNA expression, copy number variation, DNA methylation and miRNA) is high-dimensional itself. When conducting integrative analysis, the data aggregated from different levels are of even higher dimension. Among the high dimensional omics features, only a small subset of them have important implications [57]. Consequently, variable selection plays a critical role in the search of such a subset of features for integrating the multi-omics data.

A seemingly straightforward integration strategy, which turns out to be surprisingly effective sometimes, is to treat omics measurements from different platform equally and perform integration in a parallel fashion. As the omics measurements profiled on different platforms are interconnected (for example, the cis-regulation effect), a popular trend nowadays is to incorporate the regulatory effect by conducting hierarchical integration. Most of the integrated analysis (via variable selection) can be grouped according to the two strategies.

### 3.1. Parallel Integration

If an outcome variable is available, traditional single-level analysis investigates the association between individual molecular levels and a phenotype of interest separately. Parallel integration treats each type of omics measurements equally. Supervised integration with parallel assumptions can be viewed as a direct extension from single-omics data analysis to integrative analysis, where important associations between multi-level omics measurements and the same outcome, such as cancer survival, have been identified simultaneously in a joint model. The scheme of parallel integration is shown in Figure 1. Below we first review parallel integrated analysis in cancer prognostic studies.

#### 3.1.1. Supervised Parallel Integration

A comprehensive study is the integration of TCGA data in Zhao et al. [58], where four types of genomic measurements, mRNA expression, copy number variations, DNA methylation and microRNA, plus the clinical data, have been integrated in a parallel manner for four types of cancers (AML, BRCA, GBM and LUSC) collected by TCGA. LASSO, PCA and PLS (Partial Least Square) have been adopted to assess the prediction performance of parallel integration under survival outcomes. Denote *Y* as the cancer prognosis. Let *T* be the event time and *CT* be the corresponding censoring times, then we observe *Y* = (min(*T*,*CT*), *I*(*T* ≤ *CT*)). With the cancer outcome, the model is $Y\sim C+X^{1}+X^{2}+X^{3}+X^{4}$, where $X^{m}$ denotes the *m*th level $n\times p_{m}$ omics measurements (*m* = 1, 2, 3, 4) and C is the n×q dimensional clinical and environmental covariates. Simple models combining less omics-levels have also been considered. Although no substantial improvement in prediction has been observed through integration, the higher C-statistics (thus better predictive power) of mRNA expression compared to other omics measurements does indicate its importance. Such a pattern is sensible since mRNA expression has the most direct influence on cancer outcomes and other omics measurements’ effects on the clinical outcomes are mediated by gene expressions.

Jiang et al. [59] has conducted effective integration of multi-dimensional measurements on TCGA melanoma data. Elastic net, sparse PCA and sparse PLS approaches have been used to first extract important features from each type of omics dataset. Then the additive model can describe contributions from both clinical variables and all types of omics data under survival response. The most relevant variables (signals) are selected from all possible platforms. Results show that the improved prediction (corresponding to higher C-statistics) is due to the integrated multidimensional omics profiles in melanoma. In particular, methylations are included in the models with the highest predictive power under all the three approaches (Elastic net, SPCA and SPLS).

coxPath Mankoo et al. [60] has developed coxPath, a multivariate regularized Cox regression model, to combine mRNA, microRNA, CNV and DNA methylation data from TCGA ovarian cancer. As another successful application of variable selection methods in integration studies, coxPath is based on a predictor-corrector path-following algorithm for Cox proportional hazards model with LASSO penalty (Park and Hastie [61]). Important molecular features are identified from both single level data and integrated data, under progression free survival and overall survival, respectively. Before conducting feature selection, coxPath reduces the dimensionality of the omics data by using the pairwise association between mRNA expression and the other three types of omics measurements. Although this screening strategy can be viewed as an attempt to utilize the regulatory information among multi-level data, regularized selection of omics features has been carried out in a parallel fashion.

**Remarks:** The parallel assumption significantly simplifies the modelling of multi-level omics data, so integration in cancer prognostic studies can be carried out using existing popular variable selection methods such as LASSO and elastic net. As penalization methods can efficiently handle moderately high dimensional data, all the three studies perform prescreening on the original dataset to bring down the number of omics features subject to penalized selection. A supervised screening using marginal Cox regression has been adopted in [58,59] and a correlation based approach has been adopted in [60]. In the recent decades, the development of variable selection methods for ultra-high dimensional data has attracted much attention [62] and tailored methods for ultrahigh dimensional data under prognostic studies are available [63,64]. It is of much interest and significance to extend such a framework to the multi-dimensional omics data.

#### 3.1.2. Unsupervised/Semi-Supervised Parallel Integration

While the parallel integration strategy has been adopted in the above prognostic studies with patients’ survival as the clinical endpoint, it also plays an important role when the clinical outcome is not available or not of main interest, such as in unsupervised clustering studies. The penalized loss (or likelihood) function can be constructed based on the approximation loss between the projected multi-omics data matrix and the original data matrix.

When integrating omics data from two platforms, for instance, gene expression and CNVs, a natural goal is conducting canonical correlation analysis (CCA) to seek linear combinations of highly correlated GEs and CNVs for a better understanding of disease etiology, where the maximum correlation between two projected omics measurements is of particular interest. Such a correlation-based integration is tightly connected to covariance-based integration (Lê Cao et al. [56]) and co-inertia based integration (Meng et al. [65]). Another line of unsupervised integration studies is based on low rank approximation to the original omics data matrix, such as JIVE. Note that the connection between these two types of data projection methods has been pointed out in Witten et al. [66], among many other studies. Although a distinct boundary between the two does not exist, for convenience, we still group the unsupervised integration studies into the two categories.

##### Correlation, Covariance and Co-Inertia Based Integration

PMD Penalized Matrix Decomposition, a framework to compute a low rank approximation for a matrix, has been proposed in Witten et al. [66] to integrate two types of omics data measured on the same set of subjects. PMD leads to penalized CCA when the matrix is a cross-product matrix between the two data types (gene expression and copy number variation). While the L1 penalty is imposed on the canonical variate for genes, to better describe the contiguous regions of CNVs, a fused LASSO penalty is adopted for the canonical variate corresponding to CNVs. A meaningful set of genes that have expressions correlated with a set of CNVs has been identified. Witten et al. [66] has pointed out that PMD can also lead to sparse PCA under proper choices of the penalty on matrix structure. The connections among multiple work on sPCA through the bicondvexity formulation have also been demonstrated.

Extensions of PMD The sparse CCA that is developed in the PMD framework has been generalized in the following two aspects (Witten and Tibshirani [26]). First, with a clinical outcome (such as patients’ survival), a sparse supervised CCA (sparse sCCA) is formulated to seek linear combinations of omics data (from two platforms) that are highly correlated while being associated with the outcome. Second, omics data from more than two platforms can be integrated via the sparse multiple CCA (sparse mCCA). Connections between the two aspects can be established by treating outcome as the data from the third platform in sparse mCCA. Gross and Tibshirani [67] has investigated another formulation of sparse sCCA, which is termed as collaborative regression or Collre, by considering the objective function based on the prediction performance of response with respect to the data from two platforms, as well as how different the two predictions are. Convex penalty functions such as LASSO and fused LASSO are added to define the penalized collaborative regression. The ultimate goal is to discover signals that are common to the multi-dimensional data and relevant to the response. Other extensions of PMD include the canonical variate regression (CVR) [68], where the CCA criterion for the identification of canonical variates and regression criterion in terms of predictive modelling are unified with the CVR framework.

sPLS Lê Cao et al. [56] has provided a sparse partially least square (sPLS) approach to conduct simultaneous integration and variable selection on pair-wise omics datasets. Based on connection between the singular value decomposition (SVD) and PLS loadings, Lê Cao et al. [56] has developed the sPLS algorithm using SVD computation and an iterative PLS algorithm. L1 penalty has been imposed to achieve sparse estimates on loading vectors corresponding to the two omics datasets. A comprehensive comparison among sPLS, sparse CCA with elastic net penalty (CCA-EN) and Co-Inertia Analysis (CIA) on a cross-platform study has demonstrated comparable performance of the first two approaches, as well as their superiority over the CIA in terms of selecting relevant information [69].

PCIA Co-inertia analysis (CIA) is a multivariate statistical technique originated from ecological studies [70]. It can be considered as a generalization of CCA and PLS. Co-inertia is a global measure that quantifies the level of co-variability between two heterogeneous datasets. CIA explores the maximum co-variability between the two datasets and thus can be extended to pairwise omics data integration. Meng et al. [65] has generalized CIA to integrate multiple omics data. Penalized co-inertia analysis [71] has been developed to achieve sparsity and better interpretation of the results in CIA. LASSO and network-constrained penalty have been imposed separately. In particular, the network penalty helps incorporate prior biological information in penalized identification of sparse loadings. PCIA unravels sensible variables for cancer etiology when integrating the gene expression and protein abundance data from the NCI-60 cell line data.

**Remarks:** Reviewing integration studies from the variable selection point of view allows us to summarize correlation, covariance and co-inertia based methods in the same category. As we have discussed, the nature of integration characterizes the un-regularized loss function in the optimization criterion. These studies investigate the relationship among multi-level omics data and the resulting loss functions share similar formulation.

This subsection provides additional support to our remarks on the connections among feature selection, data integration and unsupervised analysis. The co-inertial analysis (CIA) examines the concordance between two sets of data in an unsupervised manner and can be adopted for omics data integration readily. So feature selection is not a necessary step for data integration and unsupervised analysis. However, as shown in Lê Cao et al. [69], choosing the subset of important features (or loadings) does improve integration performance, which connects the sparse version of unsupervised methods to variable selection and omics data integration.

##### Low Rank Approximation Based Integration

iCluster Shen et al. [72] has developed a penalized latent regression model, iCluster, for the joint modelling of multiple types of omics data in order to determine a single cluster assignment, instead of clustering each data type separately first and then conducting a post hoc manual integration. The K-means clustering has been reformulated as a Gaussian latent variable model and the connection of corresponding MLE to the PCA solution has been demonstrated. iCluster adopts the Gaussian latent variable framework and incorporates multi-platform data for integrative clustering and tumor subtype discovery. The model decomposes as X=WF+E, where X is the p×n omics measurement matrix for all the M levels. W, F and E are p×k projection matrix, k×n cluster indicator matrix and p×n error matrix, respectively. Furthermore, Shen et al. [73] has systematically investigated the penalized EM algorithm for iCluster with LASSO, Elastic net and fused LASSO penalty functions to accommodate different natures of the omics data and identify important genomic features that contribute to clustering.

iCluster+ A number of improvements has been made to iCluster to further accommodate the disparate nature of omics measurements. iCluster+ (Mo et al. [74]) incorporates a diversity of data types, including binary mutation status, multicategory copy number states (gain/normal/loss), count sequencing data and continuous data, and adopts tailored modelling strategies such as logistic regression, multi-logit regression, Poisson regression and standard linear regression, correspondingly. L1 penalty has then been considered for penalized estimation to pursue sparse identification of loadings.

iClusterBayes Recently, Mo et al. [75] has developed iClusterBayes, a fully Bayesian latent variable model for iCluster+. iClusterBayes incorporates the binary indicator prior in the iCluster framework for Bayesian variable selection and further generalizes to models with binary data and count data via Metropolis-Hasting algorithm. One limitation for both iCluster and iCluster+ is computation cost. Meng et al. [76] has proposed moCluster to speed up computation by first conducting a multiblock multivariate analysis which is then followed by an ordinary clustering analysis.

Joint Factor Analysis iCluster framework assumes a consistent clustering structure across multi-level omics data, which may not hold for some cases. Ray et al. [77] has proposed a Bayesian joint factor model to decompose the multi-level omics data matrix into a common component across all data types, a data-specific component and the residual noise. Both the common $\left(W^{c}\right)$ and data-specific $\left(W^{s}\right)$ factors have been assigned a shared set of factor loadings F as $X=\left(W^{c}+W^{s}\right)F+E$, where the sparsity in factor loadings F has been induced by the student-t sparseness promoting prior (Tipping [78]) and the sparsity in the feature space has been imposed by a beta-binomial process ([79,80,81]) on the factor scores $W^{c}$ and $W^{s}$. Two sets of joint analysis (GE and CNV, GE and DNA Methylation) on TCGA ovarian cancer have identified potential key drivers for ovarian cancer.

JIVE The Joint and Individual Variation Explained (JIVE) model (Lock et al. [27]) partitions the variations of the multi-omics data into the sum of three components: (1) a low rank approximation accounting for common variations among omics data from all platforms, (2) low rank approximations for platform-specific structural variations and (3) residual noise. Compared to the Bayesian joint factor analysis (Ray et al. [77]), JIVE considers an alternative decomposition model as $X=W^{c}F^{c}+W^{s}F^{s}+E$, where two different loading factors, $F^{c}$ and $F^{s}$, have been modelled for common factor ($W^{c}$) and platform-specific factor ($W^{s}$), respectively. JIVE uses L1 penalty to encourage sparsity in both joint and individual patterns. Coined within the PCA framework, the common and platform-specific variations are not related in JIVE, which results in superior distinction between cancer subtypes (and other biological categories) over correlation and covariance based integrative methods when analyzing TCGA GBM gene expression and miRNA data. JIVE has been extended to JIC, joint and individual clustering analysis [82], to simultaneously conduct joint and omics-specific clustering on gene expression and miRNA levels on TCGA breast cancer.

**Remarks:** JIVE models global and omic-type-specific components simultaneously. Lock and Dunson [83] extends the modelling strategy within a Bayesian framework to discover global clustering pattern across all levels of omics data and omics-specific clusters for each level of data. This approach, termed as Bayesian consensus clustering (BCC), determines the overall clustering through a random consensus clustering of the omics-type-specific clusters. The complexity of MCMC algorithm of BCC is in O(NMK) where N, M and K are sample size, number of data sources (platforms) and number of clusters, respectively. Therefore, BCC is computationally scalable especially for a large number of sample size and clusters. Extensions of BCC to the sparse version can be made by following Tadesse et al. [84].

Sparse methods, including variable selection and the sparse version of dimension reduction, are crucial for the clustering of high dimensional data [85]. However, not all the integrative clustering methods can perform clustering and variable selection at the same time, including BCC and MDI [86]. A major concern is the difficulty in implementation [87]. Instead, a subset of omics features from different platforms has been prescreened for subsequent integrative clustering analysis, such as in Kormaksson et al. [88] where the mixture model based clustering has been adopted.

### 3.2. Hierarchical Integration

On the contrary to parallel integration, hierarchical integration incorporates the prior knowledge of regulatory relationship among different platforms of omics data in the integration procedure and thus the integration methods are developed to more closely reflect the nature of multidimensional data. The integration scheme is shown in Figure 1.

#### 3.2.1. Supervised Hierarchical Integration

iBAG A Bayesian framework for the integrative Bayesian analysis of genomics data (iBAG) has been proposed in Wang et al. [89]. At the level of mechanistic model, gene expression has been decomposed into two components, one component directly regulated by its regulator DNA methylation and the other component influenced by other mechanisms. The associations between patients’ survival and the two components of gene expressions have been modelled in the clinical model. The conditional Laplace prior in Bayesian LASSO (Park and Casella [34]) has been adopted for Bayesian regularization and variable selection, and Gibbs sampling has been conducted to identify multiple relevant methylation-regulated genes associated with patients’ survival.

LRMs Zhu et al. [90] has developed linear regulatory modules (LRMs) to describe the regulation among different platforms of omics data. Through penalized singular value decomposition, the incorporation of regulatory relationship between sets of GEs by sets of regulators significantly differs from existing approaches. Moreover, the regulated GEs, residual GEs and residual regulators are all included in the clinical outcome model and important markers have been identified by penalization. Using DNA methylation and CNVs as the sets of regulators, LRMs leads to improved prediction performance in terms of C-statistics, compared to other methods including the ones ignoring the regulatory information.

ARMI Assisted Robust Marker Identification [91] is a robust variable selection method to integrate gene expression and its regulators while considering their regulatory relationship. The robust least absolute deviation (LAD) loss is adopted to accommodate heavy-tailed distribution of the clinical outcome. In particular, ARMI is formulated based on two separate regularized regressions, one on GEs and the other one on its regulators, for the response variable, as well as regularizing the difference between the two (GE and regulators) coefficient vectors to promote similarity. While ARMI is related to collaborative regression reviewed in parallel integration, it significantly differs due to the spirit of hierarchical integration and robust penalized identification.

Besides the above hierarchical integration studies, the strategy has also demonstrated effectiveness in investigating the association among (epi)genetic associations.

remMap To examine the alternations of RNA transcript levels due to CNVs, Peng et al. [92] model the dependence of GEs on CNVs (both are of high-dimensionality) via sparse multivariate linear regressions. Motivated by the existence of master predictors (CNVs) that regulates multiple GEs and correlations among the predictors, remMap utilizes a combination of L1 and L2 norms as the penalty functions to select the master predictors while promoting the network structure. An indicator matrix of prior knowledge has been incorporated in penalized selection to avoid shrinkage on established regulatory relationship between the predictor (CNV) and multiple responses (GEs). In the analysis of breast cancer data, a trans-hub region has been identified.

Robust Network A newly developed robust network-based methods for integrating (epi)genetic data (Wu et al. [93]) has been motivated from the following observations in data analysis. First, many GEs have shown heavy-tailed distributions and non-robust (such as least square based) methods may yield biased estimation. Second, the effect of cis-acting regulation of CNV on GEs can be non-linear. Wu et al. [93] accommodates the heavy-tailed distribution through LAD loss function and the nonlinear cis-acting regulation through a partially linear modelling approach. In addition, a network-constrained penalty term has been included for the correlation among CNVs. The computation has been efficiently achieved by coupling iterative MM step with coordinate descent framework. A case study on the TCGA melanoma data has revealed the superior performance in both prediction and identification.

#### 3.2.2. Unsupervised Hierarchical Integration

While the hierarchical modelling strategy has demonstrated effectiveness in the supervised integrative analysis where an outcome (such as patients’ survival and epigenetic measurements) is of interest, it also gains popularity in unsupervised integration, especially the clustering analysis.

Assisted Clustering Hidalgo and Ma et al. [94] has proposed a two-stage framework to conduct integrative clustering analysis on gene expressions. First, important GE-CNV relationship has been identified through elastic net where the correlations among regulators (such as CNVs) can be properly accounted for. Then, the assisted NCut measure incorporating weight matrices corresponding to both original and regulated GEs is adopted to cluster GEs. Such a two stage framework has been extended in Hidalgo and Ma [95] for the simultaneous clustering of multilayer omics measurements. Specifically, a three-layer Protein-GE-CNV structure has been of main interest. At the first stage, elastic net has been adopted to identify sparse regulatory relationship between GEs and CNVs and between Proteins and GEs. Next, a multilayer NCut (MuNCut) criterion has been proposed to incorporate both within platform and across platform information for the clustering of all the three types of omics measurements simultaneously. Case studies in both work have demonstrated the advantage of assisted clustering strategy.

GST-iCluster Group structured tight iCluster has been developed in Kim et al. [96] to improve the variable selection performance of iCluster [72]. Feature modules defined according to gene symbol annotation consist of multi-level omics features, which have been incorporated into the penalized identification of cluster assignment in iCluster framework. The overlapping group LASSO penalty has been adopted to account for the overlapping features in different modules. In addition, for a given sample, if all the latent variables are 0, the sample is discarded from clustering to encourage tight clusters. While the overlapping group LASSO helps incorporate regulatory flows among different levels of omics data in clustering, the tight clustering improves interpretation and reproducibility of clustering analysis.

IS-*K* means Huo and Tseng [97] has developed an integrative sparse K-means approach with overlapping group LASSO to perform omics feature selection and cancer subtype identification. The formulation of feature group is flexible, which can be the ones from multi-level omics data (such as mRNA, CNV and Methylation) with the same cis-regulatory information or from the pathway-guided clustering scenario. Within the sparse K-means framework, the overlapping group LASSO has been reformulated as a constrained optimization problem, which can be efficiently solved via ADMM (Boyd et al. [98]). Applications on multi-omics data from breast cancer and leukemia demonstrate improved performance in terms of identification and functional annotation of important omics profiles, as well as accuracy of clustering analysis and cancer subtype discovery.

**Remarks:** In GST-iCluster and IS-*K* means, the feature module that consists of multi-level omics profiles has been defined to incorporate prior knowledge of regulatory mechanism in penalized identification. Assisted clustering adopts a two-stage strategy to first identify regulatory mechanism and then conduct clustering analysis based on modified Ncut measure. The two types of integrative clustering strategies differ significantly in how the regulation among multi-tier omics measurements are incorporated. However, both utilize variable selection as a powerful tool to include the regulatory information. It is worth noting that as long as appropriate similarity measures can be generated, penalization approach is not necessarily the only way to seek for regulation among different levels of omics data in assisted clustering [99,100]. Nevertheless, this approach has been shown to be very effective to describe sparse regulation in multiple studies.

### 3.3. Other Methods for Integrating Multi-Omics Data

So far, we have mainly concentrated on studies using variable selection methods to conduct omics data integration. Other statistical methods have also been developed for such a task. For example, Yan et al. [25] has carried out a comprehensive comparison of classification algorithms for integrating multi-level omics data. Multiple kernel- and graph-based methods have been included, such as support vector machine (SVM), relevance vector machine, Ada-boost relevance machine and Bayesian networks. These techniques have deeply rooted in variable selection although they are not explicitly interpreted as selecting important omics features. Among them, SVM can be viewed as a penalization method with hinge loss plus the ridge penalty [101].

We acknowledge that there are integration studies where variable selection techniques are not adopted. Since they are not of the main interest of this review, we do not include them here.

### 3.4. Computation Algorithms

Efficient and reliable computational algorithms are the key to the success of multi-omics integration studies. For penalized variable selection methods, coordinate descent (CD) is one of the primary computational frameworks for high dimensional data, where the penalized loss function is optimized with respect to one predictor, that is, the omics feature, at a time until convergence. First order methods, including gradient-, sub gradient- and proximal- gradient based methods, can be readily coupled with CD to solve a large family of optimization problems, including both robust and non-robust loss functions with convex and more complicated penalty functions [90,91,92,93]. Besides, ADMM (Boyd et al. [98] has been another major computational framework for high dimensional data. It has rooted in the dual ascent and augmented Lagrangian methods from convex optimization, yet combines the strength of both. ADMM can handle multiple constraints in optimization, which is of great importance in integrating multi-level omics data as the complex data structure and the way of conducting integration can be modelled by imposing constraints to the objective function [68,97]. In addition, EM algorithm plays an important role in traditional clustering analysis, such as the K-means clustering since whether a sample belongs to a certain cluster can be treated as a missing data problem. With the multi-omics data, penalized EM algorithms have been developed to perform both clustering analysis and variable selection. Representative studies include iCluster [72] and its follow-up studies [73] and [74]. MCMC has been the main algorithm for conducting Bayesian integrative analysis. Ideally, full conditional distributions can be derived based on proper choices of prior distributions. Then a Gibbs sampler can be constructed for fast computation. For example, in Bayesian LASSO, the conditional Laplace prior on regression coefficient is critical for formulating the Gibbs sampler while inducing sparsity [34]. Metropolis Hastings sampling can be adopted if the full conditional update is not available.

Generally, a screening procedure is needed to conduct penalized selection for ultra-high dimensional data. Omics data integration has brought a unique challenge on computational methods due to the presence of multi-platform heterogeneous omics data and the demand for more complicated and tailored methods. To conduct the integration methods on a much larger scale, ADMM is promising due to its nature as a distributed optimization method. Besides, variational Bayesian methods have been proposed as a powerful alternative of MCMC to perform fast computation for large scale datasets [102,103].

**Remarks on the Choices of Variable Selection Methods for Multi-Omics Data Integration:** Although variable selection methods have been extensively developed for integrating multi-level omics data, their connections with integration studies have not been thoroughly examined. As pointed out by one of the reviewers, “It is not necessarily immediately apparent even to those using the methods that variable selection plays a dominant role.” In this review, we have made it clear. The formulation of “unpenalized loss function + penalty function” offers a new angle of investigating integrative analysis from the variable selection point of view. The nature of integration studies characterizes the loss function, which may pose certain constraints on choosing penalty functions. For example, to robustly model the association between disease phenotype and omics features, robust loss functions, such as LAD function, have been considered. Then penalty functions of the L1 form is preferred for computational conveniences [91,93].

The choices of penalty functions are also dependent on the omics data structure. To account for more complex data structures such as spatial dependence, network structure and other types of structural sparsity among features, penalty functions beyond the baselines have been developed and adopted. For example, fused LASSO has been proposed to accommodate the strong spatial correlation along genomic ordering in CNV [30] and has been adopted in multiple integration studies [26,66,67,73]. Elastic net has also been adopted for highly correlated features [73]. The network-constrained penalty, in both L1 and quadratic forms, have been adopted to describe correlation among omics features with LAD and least square type of loss functions, respectively. Penalized estimation can also be conducted in an unsupervised and semi-supervised manner, where sparse loadings are of great interest in the low rank approximation to the original multi-omics data matrices.

We summarize existing penalization methods in Table 2 to provide some insights on their applications in integrating multi-level omics data.

### 3.5. Examples

So far, we have attempted to make the connections between integrative analysis and variable selection clear. In this section, we describe three case studies of integrating multi-omics data as shown in Table 3. Paying special attention to variable selection methods results in some interesting findings.

Rappoport et al. [18] have performed a systematic assessment of a broad spectrum of multi-omics clustering methods on ten types of cancer from TCGA. The multi-omics features of interest are mRNA expression, miRNA expression and DNA methylation. A total of nine methods, including several variable selection methods, have been compared in terms of prediction, enrichment of clinical labels and computational time. MCCA [26], multiple canonical correlation analysis, which is a penalization method, has the best prediction performance under prognosis. The rMKL-LPP [104], regularized multiple kernel learning for dimension reduction with locality preserving projections, leads to the largest number of significantly enriched clinical parameters over all ten types of cancers. Although feature selection is not explicitly involved in this method, the regularization (or penalization) has been widely adopted for variable selection methods, as we point out in Section 3.3. The runtime of nine methods on ten multi-omics dataset shows there is no significant difference between methods with and without feature selection properties. Especially, MCCA has the second shortest runtime, very close to spectral clustering which is the least time consuming.

To examine the integration of omics profiles from two platforms, Pucher et al. [25] has compared the performance of sCCA [26], NMF [105] and MALA [106] on both simulated data and the TCGA BRCA GE and DNA methylation data. Feature selection has been conducted in all the three approaches, however, with different manners. sCCA achieves the selection of important features (canonical weights) through regularization and MALA carries out gene feature selection through a combinatorial approach. NMF selects the important omics features into the multi-dimensional modules (md-modules) via the weight threshold. In all the three approaches, a cutoff to determine the number of selected variables is used. Overall, sCCA has the best identification performance and is the most computationally fast. This study indicates the advantage of using regularization (or penalization) as a tool for feature selection.

Tini et al. [19] has conducted integration using five unsupervised methods. It has shown that variable selection does not necessarily lead to improved accuracy in integrating multi-level omics data, especially for JIVE, although some method, such as MCCA, does benefit from such a procedure. They observe that Similarity Network Fusion (SNF) [107] is the most robust method as more omics features are integrated. Note that the other four methods are not robust. We have provided a detailed discussion on robustness of integration methods in the section of discussion. It is interesting to reexamine the influence of feature selection when the integration methods are robust.

In Section 2, we have made remarks on the connections among integrative analysis, unsupervised analysis and feature selection. Here we further demonstrate the connections. All the three case studies have focused on unsupervised integration. Feature selection is not a built-in component for some of the methods. Therefore, feature selection itself is not necessarily a must for integrating multi-omics data. However, it has been observed that sparse unsupervised methods, such as MCCA and sCCA, do benefit from feature selection which has been achieved mainly through regularization (or penalization). In a broad sense, prescreening of omics features before integration is feature selection, which is adopted before performing almost all the integrative analyses.

## 4. Discussion

In this article, we have reviewed multi-level omics data integration studies conducted especially through variable selection methods. Both supervised and unsupervised methods have been reviewed within the parallel and integration studies, respectively. As there exists a diversity of methods for integrating multi-dimensional omics data, the reviewed studies are limited and not exhaustive. Section 3.3 briefly summarizes integrative analyses not conducted using variable selection methods. We also acknowledge that the penalization and Bayesian methods are not the only variable selection methods. However, the two have proven to be successful in a large amount of integration studies. Reviewing the integrative analyses based on variable selection methods will provide us a unique perspective of summarizing published studies, which has not been done in existing reviews on omics data integration.

Our overview on published reviews of integration studies clearly demonstrates that variable selection methods have been extensively adopted and developed for multi-dimensional omics data integration studies, and none of these reviews have systematically investigated data integration from the variable selection point of view (Table 1). We have pointed out earlier that penalization methods are usually applicable on a “moderately” high dimensional level, therefore, there is an urgent need for ultra-high dimensional methods that can accommodate large scale omics data. Compared with penalization methods for analyzing single platform data, the statistical theories and convergence properties of the associated optimization algorithms have not been well established in integrative analysis, which demands much more effort in future research.

This article, together with other reviews (Table 1) also show the popularity of clustering. In integrative analysis, clustering is perhaps the most important tool to discover cancer subtypes, which is the very first step for the delivery of personalized medicine to cancer patients. Our review clearly indicates that, compared to the large amount of integrative clustering studies not relying on regulatory information, hierarchical integration has not been extensively conducted due to the challenge in how to efficiently incorporate such information when clustering multi-level omics measurements. Penalized variable selection has been demonstrated as an effective way in clustering to incorporate rich biological information essential for deciphering the complex biological mechanisms [94,95,96,97].

Our review suggests that the model based supervised integration methods have been predominantly developed for identifying important main effect of multi-omics features. In omics data analysis focusing on single level of omics measurements, such as mRNA expressions and SNPs, interactions have significant implications beyond the main effects. A typical example is the gene-environment interaction studies [117,118]. In G × E study, interactions between the genetic and environmental factors shed fresh insight on how the genetic effect are mediated by environmental exposures to affect disease risk [119]. The superiority of gene set based association analysis over marginal analysis, as shown in [120,121,122], has motivated the development of multiple variable selection methods for G × E interactions under parametric, semi-parametric and non-parametric models recently [123,124,125,126]. It is appealing to conduct integrative G × E analysis to unravel the role that interaction effects play in multi-level omics integration studies and how additional strength that G × E interactions bring to integration. The missingness in environmental and clinical covariates can be efficiently accommodated by the existing approach [127]. Such an endeavor will motivate the development of novel variable selection methods for integrative G × E interactions.

Another major conclusion from the review is the need for a systematic development of robust integration methods. In single level omics data analysis, the demand for robust penalization methods arises as outliers and data contamination have been widely observed in predictors and responses, in addition to model mis-specification ([32,128]). When integrating multi-dimensional omics measurements, heterogeneous data aggregated from multiple sources poses even higher demand for robustness. Non-robust variable selection methods, such as LASSO and its extensions, despite success, may still have limitations. For instance, it has been pointed out that the JIVE estimates for both joint and common structures are vulnerable to outliers [27]. Recently, robust JIVE has been proposed to overcome this issue by using L1 norm to measure the low rank approximation error [129]. An interesting open question is, does the equivalence between maximum correlation or covariance-based criterion and low rank approximation criterion still hold to some extent for robust integration method? Overall, although there exists several robust integration method [91,93], more development in methodology is still in urgent need.

In this review, we have focused on integration of multi-level omics data using variable selection methods. We acknowledge that this is by no means exhaustive on studies and methods related to integrative analysis. For example, metabolomics data integration is also an important component of integrating multi-level omics data. Metabolomics is an increasingly popular area that analyzes the large amount of metabolites from a system biology perspective. Integration of metabolomics data with other omics data, including transcriptomic data and proteomics, demands tailored statistical methodology [130,131,132]. Multivariate methods, such as PCA and PLS, have been adopted for integrating metabolomics data [130]. Whether variable selection improves integration accuracy is unclear and we postpone the investigation to future studies. In addition, new forms of data keep emerging, which has led to novel integration techniques. For example, imaging data has attracted much attention recently and has already been integrated with omics data [133,134]. Review of such data and related methods is beyond the scope of this paper and thus not pursued.

## Figures and Tables

**Table 1:** Reviews on Integrating Multi-level Omics Data (a partial list).

Reference	Type	Description
Richardson et al. [10]	Comprehensive	Review statistical methods for both vertical integration and horizontal integration. Introduce different types of genomic data (DNA, Epigenetic marks, RNA and protein), genomics data resources and annotation databases.
Bersanelli et al. [11]	Comprehensive	Review mathematical and methodological aspects of data integration methods, with the following four categories (1) network-free non-Bayesian, (2) network-free Bayesian, (3) network-based non-Bayesian and (4) network-based Bayesian.
Hasin et al. [12]	Comprehensive	Different from the studies with emphasis on statistical integration methods, this review focuses on biological perspectives, i.e., the genome first approach, the phenotype first approach and the environment first approach.
Huang et al. [13]	Comprehensive	This review summarizes published integration studies, especially the matrix factorization methods, Bayesian methods, network based methods and multiple kernel learning methods.
Li et al. [14]	Comprehensive	Review the integration of multi-view biological data from the machine learning perspective. Reviewed methods include Bayesian models and networks, ensemble learning, multi-modal deep learning and multi-modal matrix/tensor factorization.
Pucher et al. [15]	Comprehensive (with case study)	Review three methods, sCCA, NMF and MALA and assess the performance on pairwise integration of omics data. Examine the consistence among results identified by different methods.
Yu et al. [16]	Comprehensive	This study first summarizes data resources (genomics, transcriptome, epigenomics, metagenomics and interactome) and data structure (vector, matrix, tensor and high-order cube). Methods are reviewed mainly following the bottom-up integration and top-down integration.
Zeng et al. [17]	Comprehensive	The statistical learning methods are overviewed from the following aspects: exploratory analysis, clustering methods, network learning, regression based learning and biological knowledge enrichment learning.
Rappoport et al. [18]	Clustering (with case study)	Review studies conducting joint clustering of multi-level omics data. Comprehensively assess the performance of nine clustering methods on ten types of cancer from TCGA.
Tini et al. [19]	Unsupervised integration (with case study)	Evaluation of five unsupervised integration methods on BXD, Platelet, BRCA data sets, as well as simulated data. Investigate the influences of parameter tuning, complexity of integration (noise level) and feature selection on the performance of integrative analysis.
Chalise et al. [20]	Clustering (with case study)	Investigate the performance of seven clustering methods on single-level data and three clustering methods on multi-level data.
Wang et al. [21]	Clustering	Discuss the clustering methods in three major groups: direct integrative clustering, clustering of clusters and regulatory integrative clustering. This study is among the first to review integrative clustering with prior biological information such as regulatory structure, pathway and network information.
Ickstadt et al. [22]	Bayesian	Review integrative Bayesian methods for gene prioritization, subgroup identification via Bayesian clustering analysis, omics feature selection and network learning.
Meng et al. [23]	Dimension Reduction (with case study)	Review dimension reduction methods for integration and examine visualization and interpretation of simultaneous exploratory analyses of multiple data sets based on dimension reduction.
Rendleman et al. [24]	Proteogenomics	This study is not another review on the statistical integrative methods. Instead, it discusses integration with an emphasis on the mass spectrometry-based proteomics data.
Yan et al. [25]	Graph- and kernel-based (with case study)	Graph- and kernel- based integrative methods have been systematically reviewed and compared using GAW 19 data and TCGA Ovarian and Breast cancer data in this study. Kernel-based methods are generally more computationally expensive. They lead to more complicated but better models than those obtained from the graph-based integrative methods.
Wu et al. [present review]	Variable Selection based	This review investigates existing multi-omics integrating studies from the variable selection point of view. This new perspective sheds fresh insight on integrative analysis.

**Table 2:** Published multi-omics Integration studies using penalization methods (a partial list).

Method	Formulation	Data	Package
Sparse CCA [66]	PMD + L1 penalty PMD + fused LASSO	comparative genomic hybridization (CGH) data	PMA
Sparse mCCA [26]	CCA criteria + LAASO/fused LASSO	DLBCL copy number variation data	PMA
Sparse sCCA [26]	Modified CCA criteria + LASSO/fused LASSO	DLBCL data with gene expression and copy number variation data	PMA
Sparse PLS [56]	Approximate loss (F norm) + LASSO	Liver toxicity data, arabidopsis data, wine yeast data	mixOmics
CollRe [67]	Multiple least square loss + L1 penalty/ridge/fused LASSO	Neoadjuvant breast cancer data with gene expression and CNV	N/A
PCIA [71]	Co-inertia-based loss + LASSO/network penalty	NCI-60 cancer cell lines gene expression and protein abundance data	PCIA
iCluster [72]	Complete data loglikelihood + L1 penalty	Lung cancer gene expression and copy number data	iCluster
iCluster [73]	Complete data loglikelihood + L1 penalty/fused LASSO/Elastic Net	Breast cancer DNA methylation and gene expression data	iCluster
iCluster+ [74]	Complete data loglikelihood + L1 penalty	(1) CCLE data with copy number variation, gene expression and mutation (2) TCGA CRC data with DNA copy number promoter methylation and mRNA expression	iClusterPlus
JIVE [27]	Approximation loss + L1 penalty	TCGA GBM data with gene expression and miRNA	r.JIVE
LRM [90]	Approximation Loss (F norm) + L1 penalty	TCGA	Github *
ARMI [91]	Multiple LAD loss + L1 penalty	(1) TCGA SKCM gene expression and CNV (2) TCGA LUAD gene expression and CNV	Github *
remMap [92]	Least square loss + L1 penalty + L2 penalty	Breast cancer with RNA transcript level and DNA copy numbers	remMap
Robust network [93]	Semiparametric LAD loss + MCP + group MCP + network penalty	TCGA cutaneous melanoma gene expression and CNV	Github *
GST-iCluster [96]	Complete data loglikelihood + L1 penalty + approximated sparse overlapping group LASSO	(1) TCGA breast cancer mRNA, methylation and CNV (2) TCGA breast cancer mRNA and miRNA	GSTiCluster
IS K-means [97]	BCSS + L1 penalty	(1) TCGA breast cancer mRNA, CNV and methylation (2) METABRIC breast cancer mRNA and CNV (3) Three leukemia transcriptomic datasets	IS-Kmeans

Note: * The corresponding authors’ Github webpage.

**Table 3:** Summary of case studies from published reviews (a partial list).

Reference	Methods Compared	Dataset	Major Conclusion
Rappoport et al. [18]	K-means; Spectral clustering; LRAcluster [108] PINS [109] SNF [107,110] rMKL-LPP [104] MCCA [26] MultiNMF [105,111,112,113,114,115] iClusterBayes [75]	TCGA Cancer Data: AML, BIC, COAD, GBM, KIRC, LIHC, LUSC, SKCM, OV and SARC	MCCA has the best prediction performance under prognosis. rMKL-LPP outperforms the rest methods in terms of the largest number of significantly enriched clinical labels in clusters. Multi-omics integration is not always superior over single-level analysis.
Tini et al. [19]	MCCA [26] JIVE [27] MCIA [65] MFA [116] SNF [107]	Murine liver (BXD), Platelet reactivity and breast cancer (BRCA).	For integrating more than two omics data, MFA performs best on simulated data. Integrating more omics data leads to noises and SNF is the most robust method.
Pucher et al. [25]	sCCA [26] NMF [105] MALA [106]	The LUAD, the KIRC and the COAD data sets	For pairwise integration of omics data, sCCA has the best identification performance and is most computationally efficient. The consistency among results identified from different methods is low.

## Abbreviations

ADMM	alternating direction method of multipliers
AML	acute myeloid leukemia
ARMI	assisted robust marker identification
AUC	area under the curve
BRCA	breast cancer dataset
BXD	murine liver dataset
CCA	canonical correlation analysis
CD	coordinate descent
CIA	co-inertia analysis
CNV	copy number variation
COAD	colon adenocarcinoma
EM	expectation–maximization
GBM	glioblastoma
GE	gene expression
GWAS	whole genome association study
JIVE	the joint and individual variation explained
KIRC	kidney renal clear cell carcinoma
LAD	least absolute deviation
LASSO	least absolute shrinkage and selection operator
LDA	linear discriminant analysis
LIHC	liver hepatocellular carcinoma
LPP	locality preserving projections
LRMs	linear regulatory modules
LUSC	lung squamous cell carcinoma
MALA	microarray logic analyzer
MCCA	multiple canonical correlation analysis
MCIA	multiple co-inertia analysis
MCMC	Markov chain Monte Carlo
MCP	minimax concave penalty
MDI	multiple dataset integration
MFA	multiple factor analysis
NMF	non-negative matrix factorization
OV	ovarian cancer
PCA	principle component analysis
PINS	perturbation clustering for data integration and disease subtyping
PLS	partial least squares
rMKL	robust multiple kernel learning
SARC	Sarcoma Alliance for Research through Collaboration
SCAD	smoothly clipped absolute deviation
SKCM	skin cutaneous melanoma
SNF	similarity network fusion
SNP	single nucleotide polymorphism
TCGA	The Cancer Genome Atlas
